# Utility of Health Facility-based Malaria Data for Malaria Surveillance

**DOI:** 10.1371/journal.pone.0054305

**Published:** 2013-02-13

**Authors:** Yaw A. Afrane, Guofa Zhou, Andrew K. Githeko, Guiyun Yan

**Affiliations:** 1 Climate and Human Health Research Unit, Centre for Global Health Research, Kenya Medical Research Institute, Kisumu, Kenya; 2 School of Health Sciences, Bondo University College, Bondo, Kenya; 3 Program in Public Health, College of Health Sciences, University of California Irvine, Irvine, United States of America; Royal Tropical Institute, The Netherlands

## Abstract

**Background:**

Currently, intensive malaria control programs are being implemented in Africa to reduce the malaria burden. Clinical malaria data from hospitals are valuable for monitoring trends in malaria morbidity and for evaluating the impacts of these interventions. However, the reliability of hospital-based data for true malaria incidence is often questioned because of diagnosis accuracy issues and variation in access to healthcare facilities among sub-groups of the population. This study investigated how diagnosis and treatment practices of malaria cases in hospitals affect reliability of hospital malaria data.

**Methodology/Principal Findings:**

The study was undertaken in health facilities in western Kenya. A total of 3,569 blood smears were analyzed after being collected from patients who were requested by clinicians to go to the hospital’s laboratory for malaria testing. We applied several quality control measures for clinical malaria diagnosis. We compared our slide reading results with those from the hospital technicians. Among the 3,390 patients whose diagnoses were analyzed, only 36% had clinical malaria defined as presence of any level of parasitaemia and fever. Sensitivity and specificity of clinicians’ diagnoses were 60.1% (95% CI: 61.1−67.5) and 75.0% (95% CI: 30.8−35.7), respectively. Among the 980 patients presumptively treated with an anti-malarial by the clinicians without laboratory diagnosis, only 47% had clinical malaria.

**Conclusions/Significance:**

These findings revealed substantial over-prescription of anti-malarials and misdiagnosis of clinical malaria. More than half of the febrile cases were not truly clinical malaria, but were wrongly diagnosed and treated as such. Deficiency in malaria diagnosis makes health facility data unreliable for monitoring trends in malaria morbidity and for evaluating impacts of malaria interventions. Improving malaria diagnosis should be a top priority in rural African health centers.

## Introduction

Malaria is one of the most fatal infectious diseases in sub-Saharan Africa [1]. The African highlands (areas with elevation above 1500 m above sea level) where malaria used to be absent or very limited, have experienced periodic epidemics since the 1980’s, with more than 110,000 fatalities each year (WHO 2008). With the support of The Global Fund to Fight AIDS, Tuberculosis and Malaria, Presidential Malaria Initiatives, and other private foundations, intensive control measures have been initiated in many parts of Africa to reduce or eliminate the burden of the disease. These measures included large-scale distribution of insecticide treated nets, indoor residual spraying, and the use of efficacious drugs in the form of artemisinin-based combination therapy (ACT) to treat uncomplicated malaria, among others. Although these control measures appear to have been successful in reducing malaria prevalence and incidence [2]–[4], full coverage impact evaluations of the malaria control programs has been difficult to realise.

Many country programmes and donors are interested in evaluating programme impact with health facility based data, which are easily available in most countries in sub-Saharan Africa. Health facility clinical malaria data could be used to evaluate the impact of intervention such as distribution of Insecticide Treated Nets (ITN) that are distributed to individuals with donor funds, with the end point being the number of clinical malaria cases reported to the health facility. This could prove to be a cost-effective source of information; however, the reliability of this data is not unequivocal. For example, Otten *et al* [5] used health facility data as evidence that the scale up in use of long lasting insecticide nets (LLINs) and case management with ACTs reduced the burden of malaria in Rwanda and Ethiopia. The use of this health facility data was seriously challenged by Rowe and others [6]. Hospital/clinic based malaria data are suspected to be affected by serious misdiagnosis, leading to either over- or under-estimation of malaria burden. Malaria misdiagnosis is likely to impact more heavily on the poor population in the remote rural community where health clinics are poorly equipped or the nurse and clinician training is lacking [7]. It is also presumed that malaria home treatment by the community could lead to under-reporting of clinical malaria cases in hospitals..

The World Health Organization guidelines for the treatment of uncomplicated malaria recommend a parasitological confirmation of diagnosis in all patients suspected of having malaria before treating. These are also the treatment guidelines adopted by the Ministry of Health of Kenya. Diagnostic techniques for malaria under these guidelines include primarily microscopy and rapid diagnostic test (RDT). There is routine training of health care workers on these new guidelines. The move towards universal diagnostic testing of malaria is a critical step forward in the fight against malaria as it will allow for the targeted use of ACTs for those who actually have malaria. This will help to reduce the emergence and spread of drug resistance. It will also help identify patients who do not have malaria, so that alternative diagnoses can be made and appropriate treatment provided. The new guidelines will therefore help improve the management of not only malaria, but other febrile illnesses. This study investigated how the diagnosis and treatment of malaria by clinicians and nurses in health facilities in rural western Kenya affect the reliability of hospital-based malaria data, which could be used for evaluating malaria trends and impacts of malaria interventions. This study aimed to address two important questions: 1) what is the accuracy of malaria diagnosis in laboratories of health facilities in western Kenya highlands, and 2) what proportion of subjects who were presumptively treated for malaria based on clinical symptoms or patients' own request, were true clinical malaria cases?

## Materials and Methods

### Study Sites

**Figure 1:**
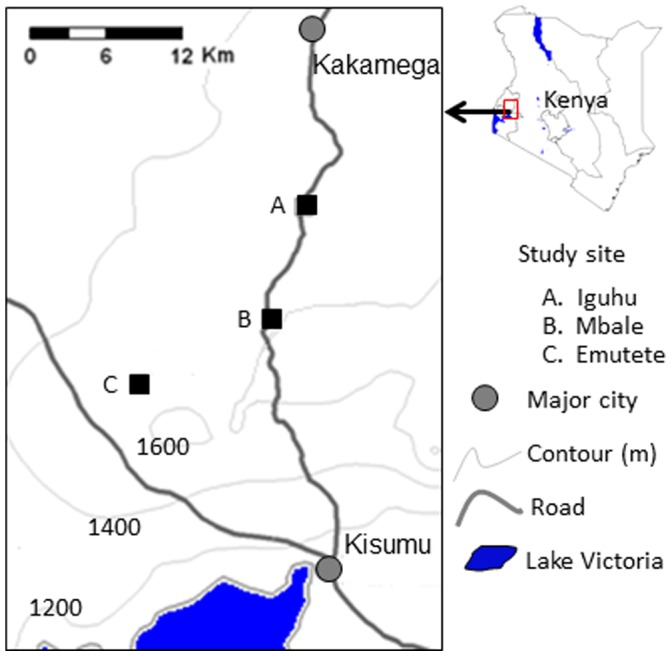
A map showing the study sites.

The study was carried out in health facilities in three districts in the western Kenya highlands. These districts were Emutete (34°66′E, 0°03′N, 1,425–1,635 m above sea level (a.s.l.) in Emuhaya district, Mbale (34°74′E, 0°07′N, 1,530–1,690 m a.s.l.) in Vihiga district, and Iguhu (34°45′E, 0°10′N, 1,430–1,580 m a.s.l.) in Kakamega district (Fig 1). Four health facilities, two being district hospitals and two being sub-district hospitals, representative of the provision of health services for residents of the communities in these districts were selected for the study. The catchment population in these health facilities ranged from 20,993–28,035 in each facility. These sites were selected because they have been previously characterized for malaria transmission patterns and vector biology [8], [9] and because they were representative of epidemic malaria situation in African highlands. The study was undertaken between July 2008 and June 2010.

### Laboratory Settings and Diagnosis of Malaria in the Health Facilities

All laboratories in each of the four health facilities had one technician in charge of diagnosis of all ailments, including malaria. Microscopy was employed in the diagnosis of all suspected malaria cases. Giemsa field stain was used to stain the blood slides before being read by microscopy. In most cases, microscope slides that had been previously used to take the blood smear of a patient could be washed and reused to take the blood smear of a new patient.

### Study Design

To check the accuracy of malaria diagnosis in the laboratories of the health facilities, we conducted an independent malaria diagnosis by collecting and re-examining the blood slides in 3,569 randomly selected subjects who had already been asked by the clinicians to take a malaria test in the health facility laboratory. The subjects were those who reported to the hospital with suspected malaria and were asked by the clinician to go for a blood test. They were recruited to the study regardless of age, sex and socio-economic status. To investigate the accuracy of the clinicians’ diagnosis of patients who were presumptively diagnosed based on clinical symptoms or patients' own request and treated for malaria, we examined blood slides from 980 new subjects who were prescribed with anti-malarial drugs by the clinicians without laboratory test.

### Data Collection

To determine the accuracy of malaria diagnosis in the laboratories of the health facilities under this study, we re-examined the thin and thick blood slides made by the laboratory of the health facilities, and compared our slide reading results with the diagnosis of the health facilities. These slides were brought to our laboratories in the Kenya Medical Research Institute in Kisumu. The thin smears were fixed in methanol and stained in 4% Giemsa for 30 min. Two certified microscopists examined the slides under ×1,000 oil immersion to identify and count the parasite species. If there were any discrepancies in slide readings, a third and more experienced technician was brought in to confirm the diagnoses. This way, we had a high quality control system in place. Our diagnostic procedures in handling the blood smears and patients were taken as the gold standard because of these quality control procedures. Parasite density was scored against 200 leukocytes when the slide was positive; otherwise, the whole slide was carefully scanned before being declared negative. A clinical malaria case was defined as an individual with fever in the presence of a positive *P. falciparum* blood smear. Fever was defined as an axillary temperature of ≥37.5°C at the time of the hospital visit or 24 hours prior to the hospital visit. Our results were compared with the diagnosis of the health facilities to determine accuracy of diagnosis of clinical malaria in the health facilities. To calculate the rate of misdiagnosis in the hospital laboratory, our slide readings were compared to that of the hospital technicians, who also employed microscopy. Our motive for making another blood slide was blinded to the laboratory technician in the hospital to ensure the objectivity of the technician’s result.

To determine the proportion of subjects who were presumptively treated as clinical malaria cases were truly infected with malaria parasite, we recruited consenting subjects at the hospital pharmacy where subjects were waiting to collect their prescribed drugs. Thin and thick smears were made and the blood slides were examined as described above. Questionnaires were given to all the subjects to obtain information on the reasons why they were presumptively treated. Additionally, a different set of questionnaires were issued to the clinicians to determine what reasons led them to decide the particular subjects received presumptive treatment without laboratory diagnosis.

### Informed Consent and Ethical Clearance

Ethical approval was obtained from the Institutional Review Boards (IRB) of the Kenya Medical Research Institute and the University of California at Irvine, USA. Written consent for adults and accent for minors were obtained from all participants.

### Data Analysis

Data from the different health facilities were pooled together since there were no significant differences between data from different health facilities. The sensitivity and specificity of the diagnoses of the clinicians in the hospitals were calculated using standard formulae, against our diagnoses which we set as the gold standard using the study malaria case definition, i.e. the presence of any parasitaemia in patients with fever after expert slide examination. Frequencies and differences between diagnosis in the lab and presumptive treatment were calculated and compared with each other using the chi-square test (χ^2^). Data analysis was stratified by age group. Cohen's inter-observer reliability kappa coefficient was used to analyse concordance between the clinic diagnosis and our gold standard lab diagnosis. Data were entered into Microsoft Excel spreadsheets processed, and analyzed using the SPSS software package (SPSS 15.0 for Windows, Chicago, IL) and JMP Statistical soft ware [10].

## Results

### Accuracy of Malaria Diagnosis in the Laboratory of Health Facilities

**Figure 2:**
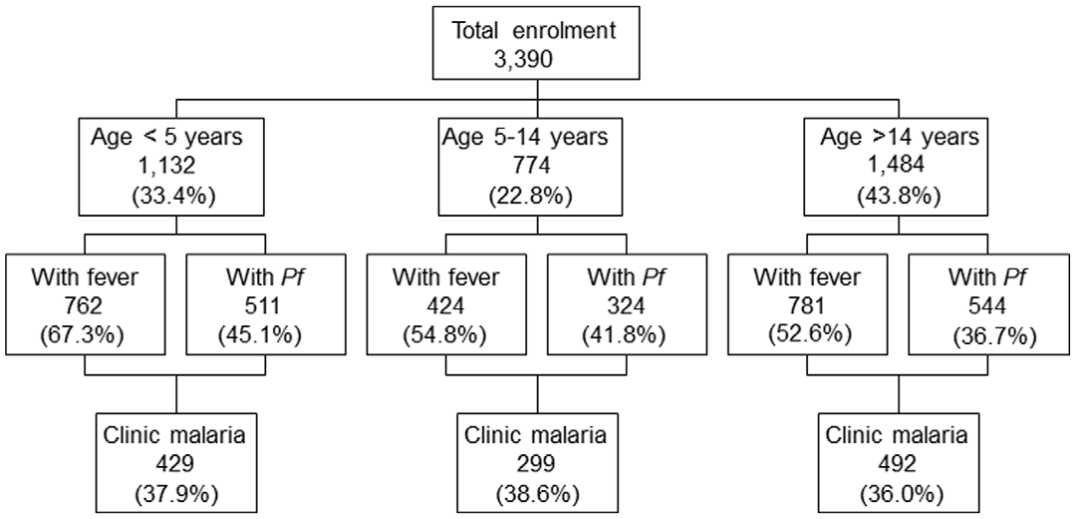
Summary of age distribution and clinical manifestation of enrolled patients sent to the labs for malaria test.

A total of 3,569 febrile subjects in four hospitals in 3 districts in Kenya were re-examined for malaria diagnosis. These subjects reported to the hospital with suspected malaria and they were asked by the clinician to go for a blood test. Among them, 179 subjects were not included in the analysis due to missing data on slide reading results by the health facility laboratory and missing temperature measurements. Out of the 3,390 patients included in the analysis, 1,132 (33.4%) were children under the age of 5 years, 774 (22.8%) were children between the ages of 5–14 and 1,484 (43.8%) were 15 years and over. The age distribution, percentage of people with fever and people who had blood smear positive results for malaria parasites, and the overall percentage of patients with clinical malaria are shown in Fig. 2.

Patients who were under the age of 5 years had the most fever cases (67.3%) compared to patients in the 5–14 year age group (54.8%) and those who were over 15 years of age (52.6%; χ^2^ = 61.2, df = 2, p<0.0001). In all age groups, less than 50% of participants had positive blood smears for malaria parasites by our team’s diagnosis, which was used as the gold standard. Parasite prevalence was 45.1% in children under the age of 5 years, with the prevalence being 41.8% in children 5–14 years and 36.7% in the over 15 year old age group (χ^2^ = 19.73, df = 2, p<0.0001). Percentage of clinical infection was 37.9% in children under 5 years, 38.6% in the 5–14 year age group and 36.0% in >15 year age group (χ^2^ = 9.31, df = 2, p<0.01).

**Figure 3:**
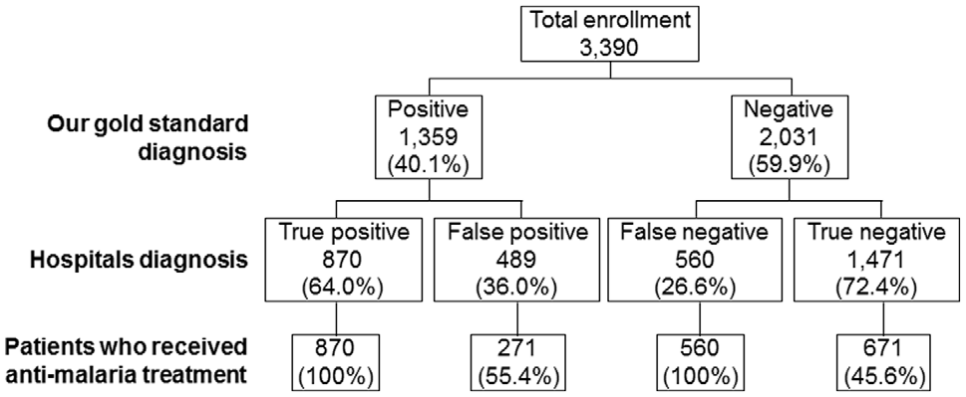
Summary of malaria diagnosis and treatment practice by the clinicians for patients sent to the lab by clinician for malaria test.

Clinical malaria was found in less than 40% of all age groups. This means that malaria was misdiagnosed in over 60% in all age groups. Yet, these people were treated with an anti-malarial. The comparison of our diagnoses to that of the clinicians’ diagnoses in the different hospitals is shown in Fig. 3. As much as 36% of patients who were diagnosed with malaria parasites in the hospitals’ labs actually did not have any parasites. Over 26% of patients who were scored negative by the clinicians actually had malaria parasites. The specificity of the diagnosis in the hospitals was 75.0% (95% CI: 30.8−35.7) and the sensitivity was 60.1% (95% CI: 61.1−67.5). There was a 67.1% concordance between the clinic diagnosis and our gold standard diagnosis.

### Presumptive Treatment by Clinicians Based on Symptoms

**Figure 4:**
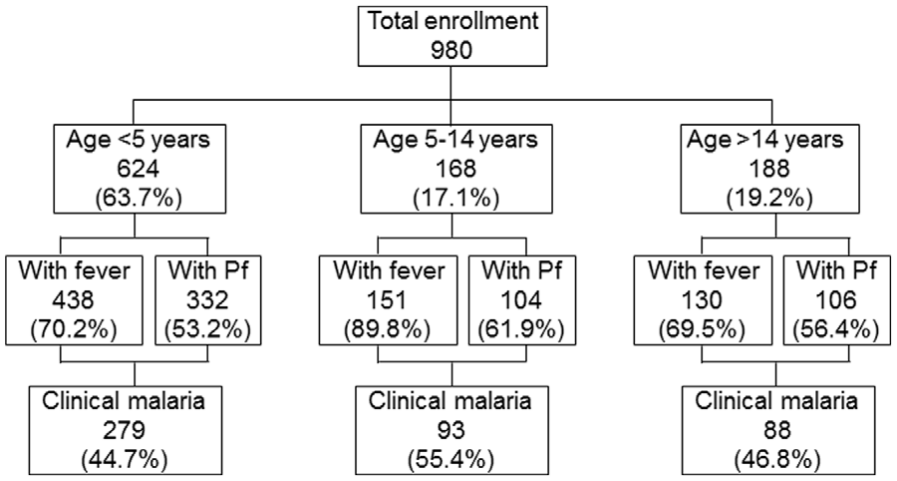
Summary of age distribution and clinical manifestation of patients presumptively treated by clinicians without a labs test for malaria.

A total of 980 new participants who were presumptively treated by the clinicians based on their symptoms in all the health facilities gave consent to our study. These were new patients who were not asked to go for a blood smear test in the lab but were treated by the clinicians based on their presenting symptoms. The age distribution of these participants, percentage of the people with fever and people who had blood smear positive results, and the overall percentage with clinical malaria are shown in Fig. 4. This included 624 (63.7%) children less than 5 years of age, 168 (17.1%) children between the ages of 5–14 years and 188 (19.2%) patients aged 15 years and above. The results of 37 patients could not be followed up and thus are not included in the analysis.

The percentage of people with fever was high among the participants with over 70% of people in all age groups having fever. The difference in numbers was however significantly different from each other. However, only 53.2% of children under 5 years of age had blood parasite positive results, whilst 61.9% and 56.4% of children between 5–14 years and participants over 14 years respectively had a positive blood smear. The difference between the participants with blood smear results in the different age categories were not statistically significant (χ^2^ = 4.16, df = 2, p>0.05). Less than half of children under 5 years and participants over 14 years actually had clinical malaria according to our gold standard definition (44.7% and 46.8%) respectively, whilst 55.4% of children between the ages of 5–14 years had clinical malaria. The statistical difference between the participants in the different age groups was significant (χ^2^ = 6.02, df = 2, p<0.05).

The decision to treat the patients presumptively was either made by the clinicians, or the patients requested that they did not want to go to the laboratory to be tested for various reasons. Among the participants, 71.6% (702/980) of them, the clinicians made the decision to treat patients presumptively, whilst 28.4% patients requested that they not go to the lab to take a test for malaria. The major reasons for patients requesting presumptive treatment instead of a lab diagnosis was lack of money (81.3%; 227/278) to pay for laboratory fees. The rest of the patients were of the view that laboratory testing was not necessary (5.2%) or they would have to queue for too long (3.5%). Lab fees for malaria diagnosis were the equivalent of US$ 0.60. However, in the lab, clinicians may want to test for other diseases, such as typhoid fever, which could increase the cost of the lab fees to between US$ 2–3.

## Discussion

In this study, we found a high proportion of misdiagnosed malaria cases from the laboratories of the health facilities. Over 60% of malaria cases were misdiagnosed. Presumptive treatment by clinicians resulted in over-treatment of many patients with expensive anti-malarials. These results revealed that health facility clinical malaria data contains cases that cannot be attributable to malaria. Furthermore, health facility clinical malaria data misses those with malaria not presenting in health facilities.

The findings of this study have brought to the forefront the need to include evaluation of hospital diagnosis and treatment practices in malaria prevention and control programmes. There is a tremendous need to improve the diagnostic capacity of health facilities in sub-Saharan Africa, which, in turn, would improve the diagnosis of clinical malaria and help to remove the reservoirs of the malaria parasite in the population. Although it is cost-effective to improve the accuracy of malaria diagnosis, simple, accurate, and inexpensive methods are not widely available, particularly in poor communities in sub-Saharan Africa where they are most needed. Health systems need to be fortified at the community level so that rapid, accurate diagnosis and effective treatment is available for those who are least able to withstand the consequences of illness [7].

The misdiagnosis and over-treatment of clinical malaria cases in health facilities in Africa, and in Kenya in particular, are not new. Several studies have reported misdiagnosed [11]–[13] and over-treated [6], [14], [15] clinical malaria cases in different countries in Africa. Ndyomugyenyi and others [14] reported in their study on peripheral health facilities in Uganda, that only 24.8% of 1627 patients had clinical malaria according to the case definition, and >75% of patients were unnecessarily treated for malaria, with few slide negative cases receiving alternative treatment. Their findings on the proportion of patients who actually had clinical malaria were even lower than ours (37%). Hume *et al* [12] also reported in a study from health facilities in Mozambique that diagnosis based solely on clinical symptoms over-diagnosed 23% of children (<16yr) and 31% of adults with malaria.

The problem of malaria misdiagnosis and over-treatment cannot be blamed entirely on the clinicians in the hospital. Clinicians might be following national guidelines, which could be derived from the WHO recommendations for treating uncomplicated clinical malaria, especially in children under the age of 5 years. The problem is also related to health system infrastructure and arrangements. Typically, in a laboratory setting in a health facility in Africa, there is enormous pressure on the technician in the health facility to produce lab diagnoses for several diseases within a relatively short time. In our study, we observed that the quality of the slides made in the health facility laboratory was poor, with not much attention given to the reading of the blood slides for malaria which are often read in a hurry.

The consequences of misdiagnosis of malaria are felt at individual, household, and national levels. Treating all fevers presumptively as malaria masks underlying and potentially fatal conditions [16]. Individuals wrongly diagnosed with malaria will be exposed to unnecessary side-effects of drugs, and the true cause of their illness will not be recognised or treated. This scenario is likely to lead to prolonged and worsening illness with loss of income or productivity, and repeated visits to health providers. Thus, investment in accurate malaria diagnosis and appropriate management at the primary level is critical for improving health outcomes and reducing poverty [7].

Both under-diagnosis and over-diagnosis of malaria cases have substantial public health implications in sub-Saharan Africa. Missing true malaria cases threatens the lives of otherwise healthy people, whereas patients wrongly diagnosed with, or treated for malaria often have different illnesses, especially bacterial diseases, some of which are potentially fatal, that are not being treated [17]. In a recent study of children reporting to health centres in Uganda, Källander and others [18] found that 30% had symptoms compatible with both pneumonia and malaria and required dual treatment. O’Dempsey *et al* [16] have concluded that community treatment of all childhood fevers as malaria is likely to result in malaria over-diagnosis with consequent under-diagnosis of other fever-causing disorders such as pneumonia and typhoid. Many infectious diseases mimic malaria and this strategy leads to high rates of over-diagnosis and over-treatment of malaria [19]. When giving an anti-malarial, the health worker is less likely to look for another treatable cause of fever, and this leads to higher morbidity and mortality due to delay in giving appropriate treatment. Over-diagnosis also leads to overuse and misuse of expensive anti-malarial drugs that are being used in many health facilities in sub-Saharan Africa. It also potentially increases the risk of the spread of drug-resistant malaria. The general public could potentially lose trust in the real efficacy of ACTs that have been deployed as first line anti-malarials [20]. The economic implications of over-diagnosis are considerable and undermine the cost effectiveness of the newer artemisinin based combination therapies (ACTs) [21].

The current WHO guidelines recommend treatment of only laboratory-confirmed cases for uncomplicated malaria. However, these are not strictly adhered to by many clinicians. Also, from our study, we report that most people did not want to be tested for malaria before being treated because they cannot afford the cost of the laboratory testing. This brings the challenge that the National Malaria Control Programmes in Africa should make testing of patients for malaria free of charge in order to adhere to the current WHO and national guidelines for treatment of uncomplicated malaria. If these guidelines are to be followed in all health facilities in Africa, this will represent a huge savings in funds for the expensive ACTs and also will contribute to the correct diagnoses of other bacterial ailments that have symptoms closer to malaria. Currently, large-scale deployment of RDTs remains a great challenge in rural African health facility settings due to lack of sustained financial mechanisms to ensure constant availability. Microscopy represents the gold standard in the diagnosis of malaria, but training, monitoring, and evaluation of the technicians performing the diagnosis should be strengthened. The rapid and accurate diagnosis of malaria is essential if effective therapy is to be successful in reducing malaria morbidity. There is an urgent need to follow the new WHO diagnostic and treatment guidelines for uncomplicated clinical malaria with the wide-scale implementation of the relatively expensive artemisinin combination therapy (ACT) and in the light of changing patterns of malaria transmission [13].

Overall, this study revealed that hospital malaria data contains cases which are not actually clinical malaria, but have been wrongfully diagnosed and treated as such. The change in malaria treatment policy from presumptive treatment to laboratory-diagnosed confirmed treatment if followed strictly should help to correct the misdiagnosed and over treated cases in all health facilities in sub-Saharan Africa. This will help turn clinical malaria data from health facilities in sub-Saharan Africa, into a more accurate assessment of the malaria burden in most communities.
